# A Dissertation on the Diseases of the Dental Pulp and Their Treatment, Prepared at the Request of, and Read before, the Associated Alumni of American Dental Colleges, at a Meeting Held at the Baltimore College of Dental Surgery, March 2, 1854

**Published:** 1854-07

**Authors:** Chapin A. Harris


*A Dissertation on the Diseases of the Dental Pulp and their Treatment*, prepared at the request of, and read before, the Associated Alumni of American Dental Colleges, at a Meeting held at the Baltimore College of Dental Surgery, March 2, 1854. By Chapin A. Harris, M. D., D. D. S.

The pulp of the tooth, from the high degree of vitality with which it is endowed, is one of the most sensitive structures of the body, and like other parts is liable to become the seat of various morbid phenomena. Its susceptibility, too, to morbid impressions, is influenced by a variety of circumstances, such as temperament, habit of body, the state of the constitutional health, the condition of the hard structures of the tooth, &c. A cause which, under some circumstances, would not be productive of the slightest disturbance, might, under others, give rise to active inflammation, together with all its painful and disagreeable concomitants. Increased irritability may exist independently of any organic change, either in the pulp, dentine, or enamel. Examples of this are often met with in females during gestation ; but it arises more frequently as a consequence of caries of the tooth than from any other cause. Even before the disease has penetrated to the central chamber of the organ, the pulp, either from functional disturbance arising from decomposition of the dentine, impaired relationship between the two, or being more exposed to the action of external deleterious agents, often assumes a most wonderful and marked increase of irritability. Impaired digestion, as well as a disordered state of other functions of the body, frequently produces the same effect.

The susceptibility of the pulp to impressions of heat and cold and of acids, is always increased by heightened irritability. When this exists to any considerable degree, the mere contact of these agents with the tooth, is often productive of severe pain, which, on their removal, usually, very soon subsides. The pulp, however, may remain in this condition for months, and even years, without becoming the seat of inflammatory action.

Preternatural sensibility of the dentine,* whether in a sound or partially decomposed state, augments very appreciably, the irritability of the pulp. Impressions of heat and cold conveyed through the conducting medium of a metallic filling, or of a thin covering of dentine, as sometimes happens when a considerable portion of the tooth has been worn away, is also a very frequent cause of heightened irritability of the pulp. With its susceptibility thus increased, the impressions produced by these agents are often a source of irritation, and even of inflamation and suppuration, causing the death of the

---

* The sensibility of dentine is sometimes so much increased that the mere contact of a hard substance with a part which has become exposed by the destruction of a portion of the enamel, is often productive of severe pain. Examples of this sort are often met with in teeth affected with caries, before the animal frame-work has suffered complete disorganization, the earthly salts only having been decomposed—thus showing that vitality often continues long after the commencement of the disorganizing process of the disease.

entire crown and inner walls of the root of the tooth. At other times, the irritation is only followed by a slight increase of vascular action and an effusion of plastic lymph over the affected part of the pulp, which is gradually converted, first into *callus* and then into *bone;* thus an additional barrier is interposed between it and the irritating agents.

The entire pulp, when the irritation, instead of being confined to the portion of its surface first affected by the irritating agent, extends to every part of it, sometimes undergoes ossification, or rather, is converted into *osteo-dentine.* The effusion of lymph, and its conversion into callus and bone is an operation which, until recently, was supposed to be wholly confined to true osseous structure, and although some one or two examples are on reeord, in which something very analogous to it had taken place, the occurrence was regarded rather as an accidental circumstance—as an exeption to the rule, and not as the result of an established law of the economy. One of the cases to which I refer is described by Dr. E. Baker, of New York, in the first volume of the American Journal of Dental Science. It consists in the union of the fractured extremities of a central incisor of the upper jaw, broken at the neck—the crown forming a right angle with the root, it having, by the accident, which occurred at about the eighth year of age, been thrown back towards the roof of the mouth, in which position it had been permitted to remain until an ossified union, so to speak, had taken place.

Now, it is evident from the appearance of the tooth, as shown by the engraving accompanying the description, that ossification of the elongated portion of the pulp had only proceeded far enough for the formation of two or three concentric layers of dentine around its peripheral surface, and although these were fractured, the crown of the tooth was retained in its abnormal position, doubtless, by the remainder of the pulp, which could not have been severed. The deposition of earthly salts, therefore, was still carried on, and the solidification of the remaining portion, while bent to a right angle, would account for the relationship maintained between the two parts of the tooth. If its investing membrane were not actually ruptured, it must have sustained some injury when the dentine, already formed, was fractured. Inflammation must have supervened, but this, as would seem from the appearance of the engraving, only caused an effusion of lymph, which, by its conversion into callus and osseous tissue, served to complete the reparative operation. Whether an *actual* union took place between this new formed tooth-substance and the fractured edges of the dentine, I am unable to say, not having had an opportunity of examining the tooth. The productive manifestation, however, evidently came from the pulp—not from the dentine—and I have referred to the case for the purpose of showing that this tissue, under certain circumstances, and to a certain extent, is endowed with regenerative attributes.

The first example of an effort at reproduction of tooth-substance to which my attention was particularly called, occurred about four years ago, in a tooth into which I had placed, some months before, a temporary filling. The pulp at the time, was considerably exposed, and, fearing an unfavorable result, I introduced, after having removed the decayed parts, a filling of Hill's stopping, leaving a small vacant space at the bottom of the cavity. On removing the filling, I perceived a white protuberance occupying the place of the vacant space which had been left at the bottom of the cavity, and which, on being touched with a sharp-pointed instrument, was found to be of about the consistence of cartilage. The question, whence did this come or how was it formed? immediately suggested itself, but it was not until I had observed the same thing in several other teeth, and ascertained that it ultimately ossified, that I arrived at the conclusion that it was callus, formed from coagulable lymph, effused from the exposed surface of the pulp.

This fact being established, the conclusion seems irresistible, that nature, under certain circumstances employs the same means for the reparation of injury here, that she does in other osseous structures of the body—the only difference being, that the lymph in the one case is effused from the lining membrane or pulp of a tooth, while in the other it is poured out from the vessels of the periosteum and fractured surfaces of a broken bone.

With these few preliminary remarks, I shall proceed to notice, first, irritation of the dental pulp.

*Irritation.*—The pulp of a tooth may become the seat of severe pain when there is no inflammation in it. The slightest increase of vascular action, when this organ is in a preternaturally irritable condition, is productive of more or less irritation. The pressure of the slightly distended vessels upon the nervous filaments distributed upon it, at such times, is sufficient to cause great pain.

Impressions of heat and cold are conveyed more readily to the pulp when the dentine is in a morbidly sensitive condition, and when this is the case they produce a more powerful effect.

The remedial indications for pain in a tooth arising simply from irritation of the pulp, consist in the removal of the primary and exciting causes. When produced by impressions of heat and cold conveyed to it through the conducting medium of a metallic filling, and intervening supersensitive dentine, the filling, if the severity and continuance of the pain is such as to warrant the belief that it will give rise to inflammation, should be removed and some non-conducting substance placed in the bottom of the cavity previously to replacing it. If this is done before inflammation actually takes place, it will prevent subsequent irritation from these causes. It is worthy of remark, however, that the pain thus produced, is in proportion to the sensibility of the subjacent dentine. If this is destroyed previously to filling the tooth, their action upon the pulp will be as effectually prevented as by the interposition of a non-conducting substance. But in the application of agents for this purpose, there is danger of destroying the vitality of the pulp. The employment of them, however, is resorted to more frequently to prevent pain during the removal of caries than subsequent irritation from impressions of heat and cold.

Arsenious acid, cobalt, chloride of zinc, and the actual cautery have all been employed in the treatment of sensitive dentine, and as there is some diversity of opinion with regard to their relative efficiency as well as safety, a few remarks upon each will not be deemed irrelevant.

The use of arsenious acid for the destruction of the vitality of the pulp of a tooth was first brought to the notice of the dental profession generally by Dr. S. Spooner, of New York, in 1836, but it had been previously employed for three or four years by his brother, Dr. J. R. Spooner, of Montreal. The promulgation of the discovery brought it almost immediately into general use. It was eagerly seized upon by the ignorant and unscrupulous, with a view to individual notoriety, and for several years almost every newspaper contained the advertisement of some pseudo-dentist, proposing to cure every form and variety of tooth-ache, in an almost incredibly short time. So *indiscriminately* and *injudiciously* was it used, that the benefit derived from it was more than counterbalanced by the evil that resulted from its employment. It was applied indiscriminately to teeth in which the lining membrane was exposed, and as soon as it had destroyed the vitality of the pulp, the decomposed dentine was partially or wholly removed, and the cavity in the crown filled without taking away the disorganized pulp of the central chamber and root. As might have been supposed, the putrid matter here, soon caused irritation and inflammation of the alveolo-dental periosteum at the extremity of the root, which ultimately terminated in the formation of abscess. A result so unexpected, and fraught with consequences so deleterious to the teeth to which the potent agent was applied, as well as to the contiguous parts, caused the more prudent members of the profession either to abandon its use altogether or to endeavor to ascertain if the morbid effects to which it gave rise, were not dependent upon something else than the mere destruction of the vitality of the pulp, and consequent death of the crown and inner walls of the root of the tooth. That there were, seemed to be rendered probable by the fact that in those cases where the pulp had been destroyed and removed by mechanical means, they were rarely developed.

Some supposed that the action of the arsenic extended beyond the extremity of the root and so impaired the vital functions of the peridental membrane, as to prevent it from furnishing the necessary supply of nutrition and living principle to the peripheral walls of the root; in consequence of which, the tooth, to some extent, at least, became a foreign body, and, acting as an irritant, gave rise to the morbid effects which followed. In this opinion, I at first participated, but subsequently, in a conversation with Dr. Maynard, this gentleman expressed to me the belief that they were dependent upon irritation produced by the disorganized matter contained in the central chamber of the tooth, and that by removing the pulp after destroying its vitality, and filling this, to the extremity of the root, they would, in a large majority of the cases, be prevented. He had at this time made the experiment in a number of cases, and the result thus far had proved highly satisfactory. It is scarcely necessary to say that the correctness of the opinion of this eminent practitioner is now very generally admitted.

But the use of arsenious acid in dental practice has not been wholly confined to the destruction of the vitality of the pulp. It was soon ascertained that the application of it to dentine would detroy its sensibility, and thus enable the operator to remove the semi-decomposed parts of a sensitive carious tooth preparatory to filling, without pain. In employing it for this purpose, however, great care is necessary to prevent the destruction of the vitality of the pulp, and the injection of the vessels of the dentine with red blood. This is very liable to happen when it is applied to a tooth of a very soft texture, especially if in the mouth of a very young person, and when the caries extends nearly to the pulp-cavity. The action of the arsenic, through the intervening hard structures, on the pulp, would seem, in the first instance, to cause, in some way or other, the decomposition of the red globules of the blood, whereby a pinkish-purple tinge is imparted to the serous portion of this fluid, which is conveyed to every part of the dentine. It seems, too, to exert some peculiar action upon the microscopic vessels of this tissue, for the fluid which they circulate is now evidently everywhere effused from their coats and brought in direct contact with the earthy salts, coloring them so deeply as to impart to the crown of the tooth a pinkish or purple hue, which may be distinctly seen through the translucent enamel covering. Three or four cases in which this has happened have occurred in my own practice, one of which I will describe.

Soon after my attention was first called to the use of arsenic in dental practice, a young lady, about fifteen years of age, applied to me to fill an upper central incisor, which, being so exceedingly sensitive as to render the removal of the decayed part almost insupportable, it occurred to me that the morbid sensibility might be removed by an application of this potent agent, having already employed it successfully several times for the destruction of the vitality of the pulps of teeth. I accordingly applied the twentieth part of a grain on a little raw cotton, previously moistened with water, closing the orifice of the cavity with yellow wax, to exclude the secretions of the mouth. My patient was now dismissed, but returned, agreeable to my request, at the expiration of four hours, when I removed the arsenic, completed the preparation of the cavity and filled the tooth.

At the expiration of about a week my patient called on me again, greatly distressed at the change which had taken place in the appearance of her tooth, every part of the crown of which had assumed a pinkish-purple hue. At first, I was disposed to believe that this peculiar appearance arose from congestion of the pulp or from sanguineous effusion from its vascular capillaries, but on removing the filling, I perceived that a reddish brown tinge had, in some way or other, been imparted to the dentine, and on perforating the floor of the cavity, the pulp, to my great astonishment, was found to have lost all vitality, and to have assumed a dark brown or purple color.

At the first meeting of the American Society of Dental Surgeons, held in the city of New York, July, 1840, I took occasion to refer to this, and some one or two other cases, in which the same effect had resulted from its application to sensitive dentine. Dr. H. H. Hayden, and, if I mistake not, some one or two others present, stated that the same thing had occurred in their practice. In a subsequent conversation with Dr. H. upon the subject, the question as to the manner in which this most singular effect was produced, having been started, I expressed the belief that it was brought about in the manner as already described. The teeth of persons who have been drowned or died of cholera or from strangulation, often present precisely the same appearance. From this, it would seem that the sudden exclusion of atmospheric air from the lungs, had the effect of causing the disintegration of the red globules of the blood, before the heart and arteries ceased to act, and hence the injection of the minute vessels of the dentine with red fluid.

But the application of arsenic to a tooth is not necessarily followed by this effect. It is only in young persons, and in teeth of a very soft texture, that it is liable to be produced, unless it is permitted to remain in the tooth a long time. When it is used merely for the purpose of destroying the vitality of the surface of the dentine at the bottom of the cavity, preparatory to the introduction of a filling, and to prevent irritation of the pulp from impressions of heat and cold, it should never be permitted to remain more than two hours. At the expiration of this time it should be removed, and after thoroughly washing and drying the cavity, the filling may be introduced, without danger of subsequent irritation of the pulp or discoloration of the tooth. The thirtieth, fortieth, or even the fiftieth part of a grain, with an equal quantity of sulphate of morphia, is sufficient to apply to a single tooth. It should be placed directly upon the bottom of the cavity, on a dossil of raw cotton or lint, moistened with water or creosote. Some prefer the latter, when the arsenic is to be applied to the pulp, but I have never been able to perceive that it possessed any advantage over the former. After the arsenic has been applied, the cavity should be carefully filled with wax, mastic, or Hill's stopping, to prevent the possibility of its escaping into the mouth and to exclude the buccal fluids. When the cavity is in the approximal surface of the tooth, additional security may be obtained by passing a ligature of floss silk three or four times around it and tying. A small ring cut from the end of a tube of caoutchouc placed on the tooth is even better than a ligature of silk. Some precaution of this kind should be used when the arsenic is applied for the destruction of the pulp, and is to remain in the tooth over night.

Professor Arthur recommends the use of cobalt for destroying morbid sensibility of dentine. He has used it for several years, and believes it to be as certain in its effects as arsenious acid, and less liable to injure the pulp of the tooth. It is the arsenic, however, with which the cobalt is combined that produces the effect, but Professor A. is of the opinion its union with this renders it less liable to be taken into the dentine by absorption, and as a consequence, less liable to produce a deleterious action upon the pulp. It is used in the form of a brownish-black oxyd, reduced to a fine powder, and applied to the tooth in the same manner as arsenious acid.

From the few experiments which I have made with it, I have not been able to discover that it possesses any advantages over arsenious acid, or that it is less liable to exert a deleterious effect on the dental pulp. The only difference which I have been able to perceive, is, that the former acts less promptly than the latter. But as I have seldom used either for the last five or six years for the destruction of morbid sensibility in dentine, I am not as competent to judge of the relative merits of the two agents as I should have been if I had had more experience in the use of the former.

For the destruction merely of morbid sensibility of the solid structures of a tooth, chloride of zinc, according to my experience, although somewhat less certain in its effects, is superior to any preparation dependent for its active properties upon the presence of arsenic. With this agent it rarely happens that more than five minutes is required to obtain the desired effect. Although a powerful escharotic, it does not, as all arsenical preparations are liable to do, produce any deleterious effect on the pulp of the tooth. When first applied it excites a sensation of heat, followed by burning pain, but these soon subside, and on removing it from the tooth, the parts of the cavity with which it was in contact, will, in a large majority of cases, be found totally insensible to the touch of an instrument. Dr. F. N. Seaburg, in the Dental Times, relates a case in which he applied it directly to the exposed pulp of an aching tooth. The pain, which at first was increased, soon subsided, and after removing the chloride, the tooth was filled in the usual way with no inconvenience to the patient.

The chloride may be applied directly to the cavity of a sensitive tooth, without being combined with any other substance, on a little raw cotton or lint, or it may be made into a paste by mixing it with an equal quantity of flour, the moisture which it absorbs from the atmosphere being sufficient for the formation of the paste, or it may be mixed with a little pure anhydrous sulphate of lime in an impalpable powder, and then applied to the tooth. But before this is done as much of the decomposed dentine as possible should be removed, and the application should be held firmly in contact with the part of the cavity upon which it is desired that it should act. This may be done by filling the cavity, after it has been put in, with softened wax or raw cotton. The chloride may remain in the tooth from five to ten minutes, or until the burning sensation produced by it ceases. A single application will generally suffice to destroy the sensibility of the walls of the cavity to a sufficient depth to enable the operator to remove any remaining portion of decayed dentine without pain, and to obtund the vitality of the floor at the bottom so much as to prevent the transmission of impressions of heat and cold to the pulp. A second, and even a third application, however, will sometimes be required.

In all cases in which I have used this agent, it has only failed in one instance to produce the desired effect, but as I rarely find it necessary to make any application to a tooth for the purpose of destroying the morbid sensibility in partially decomposed dentine, previously to removing it, and as I prefer, for the prevention of irritation of the pulp from impressions of heat and cold, placing a non-conducting substance on the bottom of the cavity, before introducing the filling, I have not used it very often. Even in the cases in which I did employ it, I was induced to do so more as a matter of experiment than from any practical advantage I hoped to derive from it.

The actual cautery was at one time much used and highly recommended by French dentists, in the treatment of sensitive decayed teeth, but the use of it was long since laid aside by American and English practitioners, as the application of it gave rise, very often, to inflammation of the pulp.

Less potent agents, such as pulverized galls, tannic acid, etc., have been employed for the purpose of destroying morbid sensibility in teeth preparatory to filling, and very often with the most gratifying results.

Having noticed the agents usually employed for destroying morbid sensibility in dentine as a means of preventing irritation, from impressions of heat and cold, of the dental pulp, I will proceed to notice a few of the non-conductors of caloric that have been used for the accomplishment of the same object. Among the substances which have been employed for this purpose, are: *asbestos*, *gutta percha*, *Hill's stopping*, which is a compound of gutta percha, carbonate of lime and some other earthy salts, *cork* and *oiled silk*.

Asbestos, one of the best-known non-conductors of caloric capable of being applied to this purpose, was first brought to the notice of the dental profession by Dr. Solyman Brown, in a paper published in the American Journal of Dental Science in 1840. At the time this article was written, the author did not claim that a sufficient number of experiments had been made with asbestos to warrant the positive assertion that its use would become general among dentists. He however thought that enough had been ascertained from the experiments which had then been made with it to render it highly probable that this would be the case, and I have no doubt that his prediction would have been verified, if another non-conducting substance, at that time unknown, which can be more conveniently applied to the cavity of a tooth, had not subsequently been discovered. As it is, it has been employed in a sufficient number of cases, to establish the fact, that the object proposed by Dr. B. can be accomplished by its use. As a non-conductor of caloric, it certainly possesses every desirable property, and it is as indestructible in a tooth as gold.

When used for that purpose, the purest variety of asbestos should be selected. A small pellet, made from the filaments of this mineral, placed in the bottom of a cavity in a tooth previously to the introduction of the gold, will effectually prevent irritation of the pulp from impressions of heat and cold. The cavity, however, should be first properly prepared, washed with tepid water and made perfectly dry. The asbestos may occupy from one fourth to one sixth of the depth of the cavity after the filling has been introduced and consolidated.

Within the last few years the concrete juice of a plant supposed to belong to the natural order *sapoteæ*, called *gutta percha*, and somewhat similar to caoutchouc, possessing great tenacity and but little elasticity, has been introduced to the notice of the dental profession. At a temperature of fifty degrees, Fahrenheit's scale, it possesses the density of wood, but at one hundred and fifty, it is plastic, and may be used for taking impressions of the mouth or be molded into any desired shape. It is soluble in chloroform, but insoluble in the secretions of the mouth.

Soon after I became acquainted with the properties of this article, I applied a drop or two of a solution of it in chloroform to the exposed pulp of a tooth which I was about to fill. The chloroform evaporated almost immediately, leaving a thin pellicle of gutta percha on the bottom of the cavity. This experiment was repeated a number of times, and soon after, I applied it to the bottom of cavities in teeth where the dentine was so sensitive as to lead to the apprehension of irritation and inflammation from the transmission of impressions of heat and cold.

The result of these last experiments was so satisfactory that I took occasion, at a meeting of the American Society of Dental Surgeons, held at Saratoga, in 1848, to call the attention of the members to the subject. But previously to this time, Dr. A. Hill, of Norwalk, Ct., had made a preparation, now generally known as "Hill's stopping," of which gutta percha forms the principal ingredient, for filling temporary teeth, and permanent teeth in cases where the sensibility of the dentine is such as to preclude the use of gold or other metallic substances. He gave me a small piece, requesting that I would make such experiments with it as I might deem necessary to satisfy myself with regard to its value. It proved more valuable than I expected, for it soon occurred to me that a thin layer of this preparation placed on the bottom of the cavity of the tooth in which the dentine was in a supersensitive condition, previously to putting in the gold, would, on account of its non-conducting properties, prevent impressions of heat and cold from being conveyed to the pulp, and as a consequence, the irritation liable to be produced by them. The result fully equalled my expectations, and I have continued to use it in cases of this sort, with the most decided advantage. It answers equally as good a purpose as asbestos, and can be applied more conveniently. It also adapts itself more perfectly to the inequalities of the floor of the cavity.

The method of applying it is very simple. The cavity being first properly prepared, a small piece of this preparation is slightly warmed by a fire, or by the flame of a candle or lamp, then placed in the bottom of the cavity and adapted to its inequalities by pressing on it gently with a large broad-pointed plugger. This done, the cavity may be filled with gold in the usual manner.

Dr. J. H. Foster, of New York, adopts a somewhat different method of procedure in the use of this compound. He first makes a cap of gold plate, fitting it accurately to the walls of the cavity. He then fills the concave part of it with this preparation, then places it in the bottom of the cavity with the convex side looking toward the orifice, and afterward fills the tooth in the usual way. His object in using the cap is to prevent depressing the floor of the cavity upon the pulp, which in those cases where it is very thin, is liable to be done, unless great precaution is used in introducing the gold. When this happens, irritation and inflammation follow as a natural consequence, and the only means of affording relief in such cases, consist in the removal of the filling, and this, unless had recourse to before inflammation takes place, will prove unavailing. When, therefore, there is reason to apprehend an occurrence of this kind, the gold should be pressed so firmly against the walls of the cavity when it is introduced, as to prevent the possibility of depressing the bottom of the cavity in the subsequent consolidating process of the extruding portion of the metal. The solidity and permanency of the filling will, of course, depend in a great degree, upon the faithfulness with which the first part of the operation is performed.

When the depth of the cavity in the tooth is sufficient to admit of the introduction of a gold cap, as recommended by Dr. Foster, without interfering with the subsequent operation of filling, there can be no possible objection to its use. Thus protected, it would be almost impossible to depress the floor of the cavity by any amount of force which might afterwards be employed in introducing and consolidating the gold. I have not, however, found it necessary, even in cases where the pulp of the tooth was actually exposed, to use it.

Cork, though an equally good non-conductor of caloric, is thought by some, as it is of a more destructible nature than asbestos or gutta percha, to be objectionable, but cut off as it necessarily would be in the bottom of the cavity beneath the filling, its liability to undergo any change, would seem to be rendered wholly impossible. The only valid objection, it seems to me, that can be urged against its use, is, that it is of a more porous nature than gutta percha, and cannot be adapted as perfectly to the inequalities of the floor of the cavity. In consequence of the former of these objections, there is danger, in introducing the filling, of forcing some portions of the gold through it, unless a very thick piece be used. Oiled silk has been used in some cases very successfully, but is not as good a non-conductor as any of the before mentioned agents.

As a means of preventing irritation and inflammation of the pulp, from impressions of heat and cold transmitted to it through the conducting medium of a metallic filling, introduced after this organ has become exposed, Dr. S. P. Hullihen described to me, in 1850, an operation which he had invented some time before, consisting in perforating the root to the central cavity with a small drill, first passed through the edge of the gum and alveolus, thus making an outlet for the escape of effused fluids and the blood which may be poured out from the vessels punctured by the point of the instrument. The operation, in the hands of Dr. H. and a few others who have practiced it, is said to have been very successful, but not having had occasion to perform it more than three or four times, since it was first made known to me, I am not able to speak from personal experience of its merits.

But a metallic filling is not the only medium through which impressions of heat and cold are conveyed to the dental pulp. When the dentine on the coronal extremity or side of a tooth becomes very thin from loss of substance, occasioned either by mechanical or spontaneous abrasion, the use of the file, erosion, or other cause, the pulp sometimes becomes painfully affected by the action of these agents. Loss of substance from any of these causes is also attended by exalted sensibility of the exposed dentine; and when this is the case, the contact of acids with it is productive of more or less pain. Nature, however, usually employs means to prevent the painful consequences that would naturally arise from continued abrasion of the coronal ends of the teeth and the consequent exposure of their nervous pulps, which consists in the gradual ossification of these organs, so that by the time they would have become exposed, they are converted into bone, or osteodentine. But this does not always take place in time to prevent irritation and pain; and when it does not, some practitioners recommend, where the loss of substance is confined to the molars and bicuspidati, the application of caps to the crowns of such teeth as have suffered most. The secretions of the mouth, however, that would necessarily accumulate between them and the teeth, would soon become vitiated and increase the already existing sensibility of the exposed dentine. The difficulty, therefore, would be greatly aggravated by appliances of this sort. The inconvenience and suffering which sometimes arise from the wearing away of the teeth, can only be prevented by avoiding the use of acids, and hot and cold beverages; and the observance even of these precautionary measures, will not always wholly prevent their occurrence.

When irritation of the pulp occurs in a tooth that has been filed on one or both sides, so much so as to leave only a thin covering of dentine, the best known means of preventing morbid sensibility of this, is to keep the filed surface constantly clean by friction, both with a brush and waxen floss silk, or some other suitable substance. This operation should be repeated after each meal, and in the morning immediately after rising and at night before going to bed.

When caries has extended to the central cavity, irritation is often produced by contact of partially decomposed portions of dentine or other foreign matter with the pulp. The proper remedial indication in such cases, it is scarcely necessary to say, consists in the removal of whatever matter from the tooth that can act either as mechanical or chemical irritants. This done, the cavity in the tooth—supposing the pulp to be in a healthy condition—should be properly filled.

But, when the irritation arises as a consequence of exalted irritability and increased vascular action of the pulp, dependent upon disease or altered function of some other part or parts of the body, the remedial indications are different. The treatment, then, should be addressed to the primary affection. Examples of this sort are of frequent occurrence. They are met with almost daily, particularly in females during gestation, and in dyspeptic individuals, and persons affected with the gout and chronic rheumatism. They are also sometimes met with in individuals who have been exposed to miasmatic emanations of marshy districts, when the irritation assumes an intermittent form, occurring at stated intervals, of twenty-four, forty-eight, or seventy-two hours, and continuing from one to three hours. Some of the worst forms of tooth-ache are produced by one or other of these causes.

The local disturbance, when it occurs in females during pregnancy, may generally be removed by mild aperients, and by warm foot-bath and anodynes at night on going to bed. When it depends upon other kinds of derangement of the uterine organs, treatment suited to the peculiar indications of the case should be instituted. When it occurs in a person affected with dyspepsia, rheumatism or gout, the constitutional treatment required by the particular disease, constitutes the proper remedial indication. When the irritation assumes an intermittent form, an emetic or cathartic, followed by quinine, will generally put a stop to the local disturbance, provided it has no connection with caries of the crown of the tooth.

But the pulp of a tooth is not only liable to *irritation*, but also to *inflammation* and a variety of other morbid phenomena. Endowed as this organ is with the most exquisite sensibility, it is keenly susceptible to the action of irritating agents of almost every kind, and therefore it is not to be wondered that it should become the seat of some of the most aggravated and painful forms of disease.

Having described some of the principal causes and treatment of irritation, I shall next proceed to offer a few remarks on inflammation.

[to be continued.]

